# Vaccination Expands Antigen-Specific CD4^+^ Memory T Cells and Mobilizes Bystander Central Memory T Cells

**DOI:** 10.1371/journal.pone.0136717

**Published:** 2015-09-02

**Authors:** Eleonora Li Causi, Suraj C. Parikh, Lindsey Chudley, David M. Layfield, Christian H. Ottensmeier, Freda K. Stevenson, Gianfranco Di Genova

**Affiliations:** Cancer Sciences Unit, Faculty of Medicine, University of Southampton, Southampton, United Kingdom; Maisonneuve-Rosemont Hospital, CANADA

## Abstract

CD4^+^ T helper memory (Th_mem_) cells influence both natural and vaccine-boosted immunity, but mechanisms for their maintenance remain unclear. Pro-survival signals from the common gamma-chain cytokines, in particular IL-7, appear important. Previously we showed in healthy volunteers that a booster vaccination with tetanus toxoid (TT) expanded peripheral blood TT-specific Th_mem_ cells as expected, but was accompanied by parallel increase of Th_mem_ cells specific for two unrelated and non cross-reactive common recall antigens. Here, in a new cohort of healthy human subjects, we compare blood vaccine-specific and bystander Th_mem_ cells in terms of differentiation stage, function, activation and proliferative status. Both responses peaked 1 week post-vaccination. Vaccine-specific cytokine-producing Th_mem_ cells were predominantly effector memory, whereas bystander cells were mainly of central memory phenotype. Importantly, TT-specific Th_mem_ cells were activated (CD38^High^ HLA-DR^+^), cycling or recently divided (Ki-67^+^), and apparently vulnerable to death (IL-7Rα^Low^ and Bcl-2 ^Low^). In contrast, bystander Th_mem_ cells were resting (CD38^Low^ HLA-DR^-^ Ki-67^-^) with high expression of IL-7Rα and Bcl-2. These findings allow a clear distinction between vaccine-specific and bystander Th_mem_ cells, suggesting the latter do not derive from recent proliferation but from cells mobilized from as yet undefined reservoirs. Furthermore, they reveal the interdependent dynamics of specific and bystander T-cell responses which will inform assessments of responses to vaccines.

## Introduction

CD4^+^ T helper (Th) cells play crucial roles in both natural and vaccine-induced immunity. Upon priming, naïve cells differentiate into distinct functional subsets with defined phenotypic and homing properties including Th1, Th2, Th17, T follicular helper, or induced T regulatory cells. Each subset appears to be tailored to exert pathogen-specific protection or immune regulation [1] [2]. Once antigen has been cleared, central memory (T_CM_) and effector memory (T_EM_) T cells remain to provide immune surveillance in lymphoid and peripheral non-lymphoid tissues respectively [3].

It is evident from human studies that natural or vaccine-induced Th_mem_ cells can persist for very long periods [4–6] but the mechanisms responsible for their maintenance remain unclear. However, pro-survival signals from the common gamma chain (γ_c_) cytokines, in particular IL-7, appear to be important [7]. IL-7 receptor signalling and expression of anti-apoptotic molecules, such as Bcl-2, promote cell survival during the T cell contraction phase and can contribute to successful effector-to-memory transition [8]. Studies in mice suggest that this transition may occur in the bone marrow, where antigen-specific CD4^+^ T cells relocate after being activated in secondary lymphoid organs. There, they down-regulate gene expression and proliferation, and survive as highly reactive memory cells in proximity to IL-7-expressing stromal cells that provide survival niches [9,10]. In humans, polyfunctional CD4^+^ memory T cells accumulate in the bone marrow in close proximity to IL-15 producing cells [11].

Previously, we investigated the dynamics of Th_mem_ cell responses to TT booster vaccination in healthy volunteers. Surprisingly, the expected expansion of TT-specific Th_mem_ cells was accompanied by an increase of Th_mem_ cells specific for two unrelated and non-cross reactive common recall antigens: purified protein derivative from tuberculin (PPD) and *Candida albicans* (*C*. *Alb*) [12]. These bystander responses had parallel kinetics to the specific response. We hypothesized that the increase of vaccine non-specific Th_mem_ cells could result from TCR-independent activation, most likely cytokine-mediated, occurring in a shared microenvironment during the vaccine-specific secondary immune response. Indeed, recent findings suggest that the cytokines produced in reactive lymph nodes can diffuse throughout the node and influence bystander cells not in close proximity to the cytokine source [13]. We demonstrated in a mouse model, that a recall response to TT can induce proliferation of previously activated CD4^+^ T cells specific for the unrelated antigen ovalbumin, proliferation being proportional to the strength of the immune responses and likely to be IL-2 mediated [14]. Another γ_c_ cytokine, IL-15, has also been shown to mediate the bystander activation and proliferation of CD8^+^ memory-phenotype T cells observed in mice, following viral infections or treatment with virus-mimetics or bacterial products [15,16].

In terms of functional outcome, it is still debated whether *in vivo* bystander activation (cytokine-mediated) of memory T cells would promote survival or lead to increased cell death. In one study, human CD4^+^ memory T cells activated *in vitro* in a bystander fashion displayed a gene expression profile distinct from antigen-specific T cells [17]. While the *in vitro*-activated bystander T cells up-regulated pro-apoptotic genes, transcripts of the pro-survival NF-kB signaling pathway were also up-regulated, making predictions of survival *in vivo* difficult. In mice, relative stability of CD4^+^ memory T cells specific for lymphocytic choriomeningitis virus has been observed following multiple heterologous virus infections, despite the parallel loss of lymphocytic choriomeningitis virus-specific CD8^+^ memory T cells [18]. Furthermore, vaccinia virus infection promoted enhanced survival of super antigen-activated T cells [19]. While conclusions on the fate of memory CD4^+^ T cells remain unclear, promotion of survival via bystander effects would be more consistent with maintenance of long-term CD4^+^ T-cell memory.

Here, we have used tetanus toxoid recall vaccination of healthy human subjects as an opportunity to probe the nature of vaccine-specific and vaccine-stimulated bystander Th_mem_.

We focused first on their differentiation stage and migratory properties, by defining their belonging to the T_CM_ and T_EM_ subsets of memory T cells [3]. Then, we addressed their survival potential, by analysing expression of IL-7Rα (CD127) which confers cells the ability to respond to the homeostatic cytokine IL-7 [8], and the levels of the anti-apoptotic molecule Bcl-2 [20]. Finally, we studied their activation status and *in vivo* proliferative activity by evaluating the proportion of CD38 and HLA-DR, and Ki-67 positive cells, respectively [21].

Our findings reveal key differences between vaccine-specific and bystander Th_mem_ cells, both increased in number in the peripheral blood following vaccination, and both sharing similar response kinetics. Whilst vaccine-specific Th_mem_ cells displayed typical features of recently generated and potentially short-lived effectors, which were still highly activated and had recently divided or were still doing so, bystander cells appeared to derive from a central memory compartment of relatively quiescent and non-proliferating cells with preserved survival potential.

## Materials and Methods

### Ethics statement

Ethical approval for the study was obtained from the Institutional Review Board and the Southampton & S.W. Hants Joint Research Ethics Committee (submission number 242/99). All subjects gave written informed consent for study participation in accordance with the Declaration of Helsinki.

### Vaccination and sample collection

Six healthy adults (3 males, 3 females, median age 32, range 25–47) received a single dose of TT vaccine (Adsorbed Tetanus Vaccine BP, Aventis Pasteur MSD) administered intramuscularly. All subjects had already been vaccinated with TT and conventional Bacillus Calmette–Guérin, but they had not received booster injections in the previous five years. Sample collection and storage was done according to our previously published protocol [12], with the exception that an additional blood sample was taken one week after vaccination in all the subjects.

### Antigens

TT not absorbed (code 02/232) and *C*. *Alb* whole extract cytoplasmic protein were purchased from the National Institute for Biological Standards and Control, NIBSC, UK. PPD prepared from human strains of *Mycobacterium tuberculosis* was obtained from Evans Vaccines Limited, UK.

### Antibody panels

The following anti-human mAbs were used: CD3 APC-Cy7 (HIT3a), CD4 Pacific Blue (OKT4), CD38 APC (HIT2), CD45RA Brilliant Violet 421 (Hl100), CD127 Brilliant Violet 421 (A019D5), CD197 (CCR7) Alexa Fluor 647 (G043H7), HLA-DR PE-Cy7 (L243) and IL-2 FITC (MQ1-17H12) purchased from BioLegend; CD4 V500 (RPA-T4), CD8 V500 (RPA-T8), Bcl-2 FITC (Bcl-2/100) and Ki-67 FITC (B56) purchased from BD Biosciences; IFN-γ PE-Cy7 (4S.B3) obtained from eBioscience and CD40L PE (TRAP 1) obtained from Beckman Coulter.

### Analysis of viable, apoptotic and necrotic cells by annexin V/propidium iodide (AV/PI) staining

To exclude the possibility of the fluctuations in T-cell responses being the result of variations in cell viability, the percentages of apoptotic and necrotic cells were analysed in both lymphocytes (effectors) and monocytes (predominant antigen-presenting population) prior to cell culture, using AV/PI staining. Briefly, PBMNC were defrosted and counted; 2.5 x 10^5^ cells were washed and re-suspended in 300μl of AV binding buffer (Biolegend). AV FITC (in house, 1.25μg) and P.I. (Biolegend, 0.22μg) were added to each tube. Cells were left 10–15’ at room temperature and then analysed on a Canto II flow cytometer. AV^neg^PI^neg^ cells were considered viable. No significant changes were found between time points; the mean percentage and standard deviation of viable lymphocytes and monocytes calculated on all subjects and on all time points were equal to 90.6% ± 4.55 and 95.2% ± 2, respectively.

### IFN-γ ELISPOT

T cell responses to vaccination were screened and evaluated using a 40h IFN-γ ELISPOT assay according to the method and criteria previously described [12]. Briefly, 2 x10^5^ PBMNC were cultured in triplicates, in RPMI-1640, L-glutamine, penicillin, streptomycin, sodium pyruvate, and 5% human AB sera (Lonza) (complete medium), in the absence (negative control) or in the presence of either TT (10μg/ml), PPD (10μg/ml), *C*. *Alb* (10μg/ml). Ag-specific responses are reported here as the number of spots per 1x10^6^ PBMNC in antigen-stimulated cultures minus the number of spots in the corresponding negative control.

### Intracellular CD40L and cytokine staining

For intracellular staining, 2 x 10^6^ PBMNCs were re-suspended in 1ml of complete medium and cultured in 15ml, 120x17mm, polypropylene tubes (SARSTEDT, Nümbrecht, Germany), in the absence (control) or in the presence of either TT (10μg/ml), PPD (15μg/ml) or *C*. *Alb* (10μg/ml). Anti-CD28 (clone CD28.2, eBioscience) was added to all tubes (1μg/ml). After 90 minutes, brefaldin A (Golgi Plug, BD Biosciences) was added (1/1000 dilution). After 6h, cells were washed and stained for surface markers first, and then they were permeabilized and stained for intracellular markers. Data were acquired on a Canto II flow cytometer and analyzed using FlowJo (7.6.5) (Treestar) software. For analysis of antigen-specific CD4^+^ T cells, between 1.5–1.8 x 10^6^ events in the singlet gate were acquired. Responses were considered positive if the frequency of the events in antigen-stimulated cultures was ≥ 0.01%, and the frequency of background events was ≤ 30% of the frequency of events in the antigen-stimulated cultures.

### Statistical Analysis

Continuous data sets were tested for normal distribution and data analysed using the non-parametric Mann Whitney U Test or independent sample T-test utilising Holm-Sidak correction for multiple comparisons. Matched pairs were analysed using Paired student T-test. Paired analysis across a time course was performed using Friedman’s test and Dunn’s multiple comparison test incorporating a multiplicity correction. Statistical analysis was performed using SPSS v21.0 (SPSS Inc,. Chicago,Il.,US) and GraphPad Prism v6.0 (GraphPad Software Inc., San Diego,Cl.,US).

## Results

### Parallel increase of vaccine-specific and bystander CD4^+^ memory T cells following TT booster vaccination

**Fig 1:**
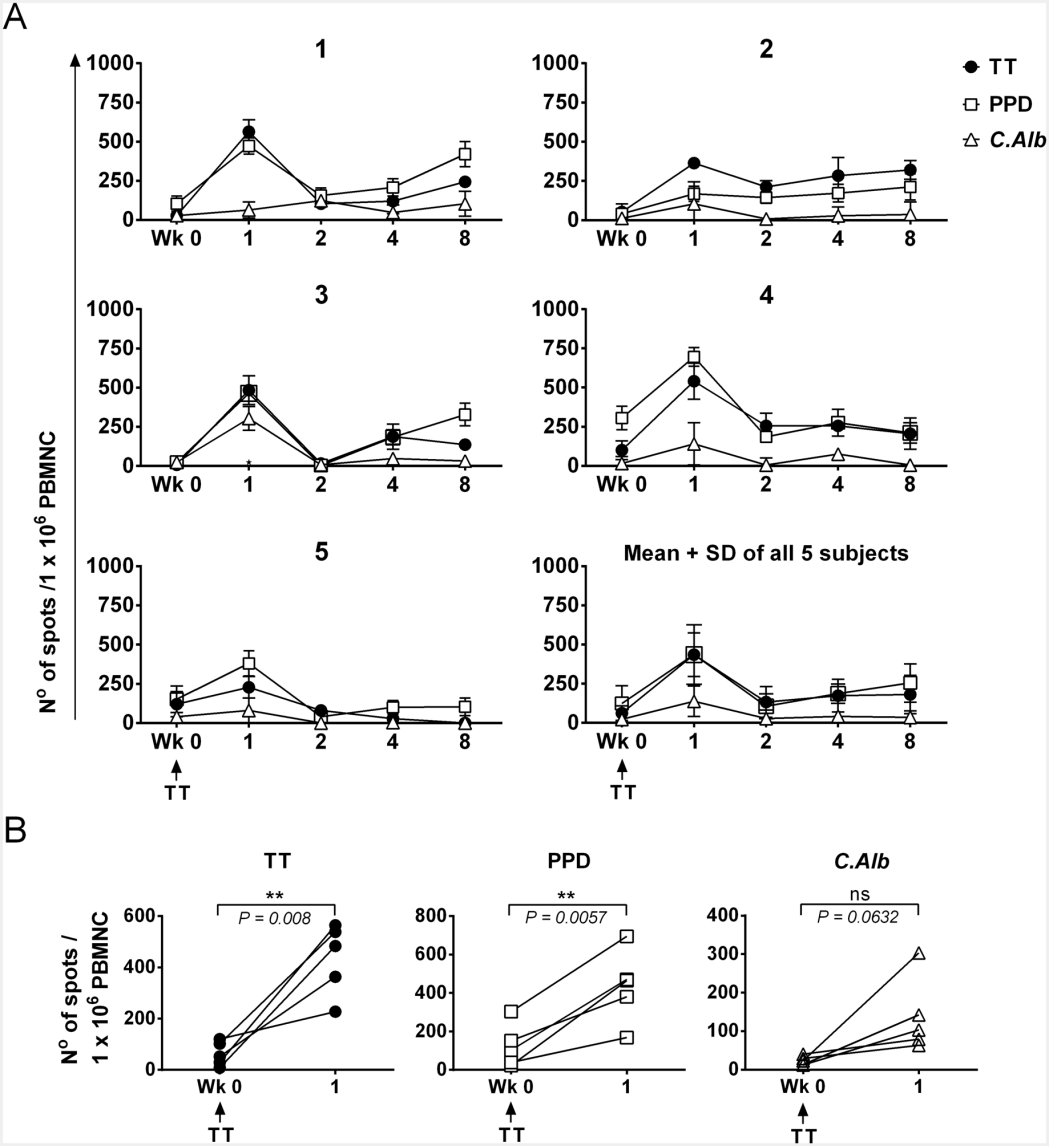
Kinetics of vaccine-specific and bystander T cell responses to TT recall vaccination. Five healthy subjects received a booster vaccination with TT and T cell responses were measured by a 40h IFN-γ ELISPOT assay, and reported as the mean number of spots ± SD of triplicate antigen-stimulated cultures, after subtracting the correspondent negative (no antigen) control. (A) Individual responses (1–5) and mean ± SD of the mean responses from all five subjects. Vaccination (Wk 0) induces increase of both vaccine-specific (TT) and vaccine-unrelated (PPD, *C*. *Alb*) IFN-γ-producing cells. Responses are highly dynamic and peak one week after vaccination. (B) Comparison between IFN-γ responses at baseline (Wk 0) and one week after vaccination (Wk 1). Vaccination induces a statistically significant increase in the number of TT-specific and PPD-specific IFN-γ-producing cells. A paired t test was applied and a two-tailed *p* value is shown.

We have previously shown how in 12 healthy volunteers a booster vaccination with TT induces expansion of vaccine-specific (TT) and an increase of bystander (PPD and *C*. *Alb*) Th_mem_ cells, with parallel kinetics [12]. Importantly, no cross-reactivity was demonstrated between TT and PPD or *C*.*Alb*. To further characterise and distinguish the vaccine-specific from the bystander response, a new cohort of 5 healthy subjects already immune to TT, received a recall TT vaccination. To confirm our previous findings, T cell responses were measured by an IFN-γ ELISPOT. The duration of the assay was optimised to allow optimal detection of cytokine response to protein antigens without induction of proliferation. Results show expansions of TT-specific T cells in all five subjects, with a peak at week 1 post-vaccination (Fig 1A). Parallel increases of IFN-γ-secreting T cells in response to PPD were evident, with similar although weaker responses against *C*. *Alb* (Fig 1A). Accordingly, pooled data demonstrated a significantly increased number of TT- and PPD-specific IFN-γ-producing cells at week 1 compared to baseline (Fig 1B). A similar trend was seen for *C*. *Alb*-specific responses, although differences failed to reach statistical significance, possibly because of the low level of detectable T-cell memory (Fig 1B). Increases were followed by contractions in both vaccine-specific and bystander responses, which then rose again in some individuals or remained stable in others. These fluctuations were not due to differential cell viability between time points (see Materials and methods) and were also seen when fresh PBMNC collected from an additional vaccinated subject, were used (S1 Fig). They rather reveal the dynamic nature of the CD4^+^ memory T cell response in humans who, in contrast to mice maintained in germ-free environment, are constantly exposed to environmental antigens.

These results confirm our previous data [12] in a new cohort of healthy subjects and set the scene for further analysis.

**Fig 2:**
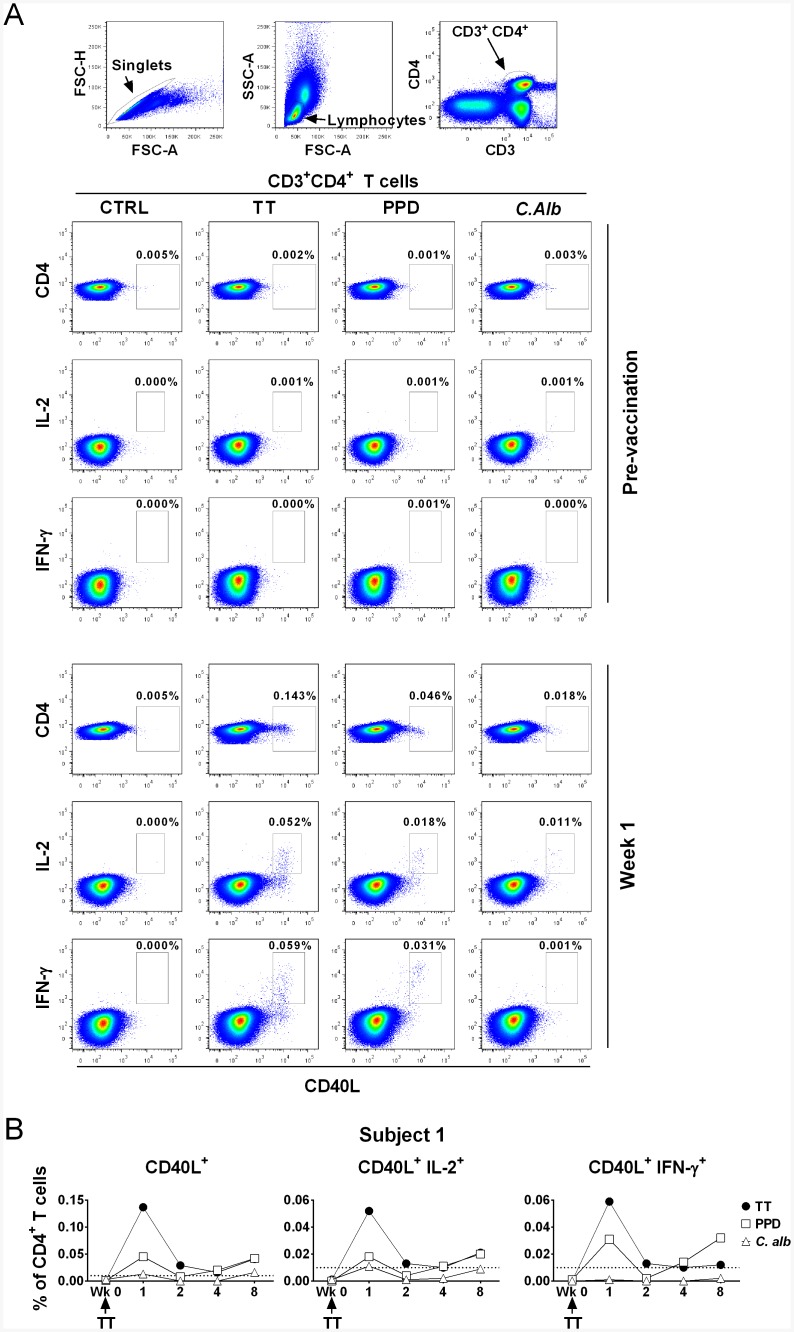
Flow cytometric analysis of vaccine-specific and bystander CD4^+^ T cell responses to TT recall vaccination detected by combined CD40L and cytokine intracellular staining. After a short term (6h) *in vitro* culture in the absence (CTRL) or in the presence of either TT (10μg/ml), PPD (15μg/ml) or *C*.*Alb* (10μg/ml), PBMNC were first stained for surface CD3 and CD4, then permeabilized and stained intracellularly with fluorescent antibodies specific for CD40L and the cytokines IL-2 and IFN-γ. (A) Data from one of the individuals (subject 1) show the gating strategy (top three dot plots) and the detection of vaccine-specific (TT) and bystander (PPD and *C*.*Alb*) CD4^+^ T cell responses before (pre-vaccination) and one week after (week 1) a booster injection of TT. Percentages indicate the frequency of positive events within the CD3^+^CD4^+^ lymphocyte population. (B) Kinetics of vaccine-specific and bystander CD4^+^ T cell responses detected by intracellular CD40L and cytokine staining in the same individual who received a booster vaccination (Wk 0) with TT. Data indicate the frequency of positive cells within the CD3^+^CD4^+^ population, obtained from the antigen-stimulated samples after subtracting the frequency of events in the control cultures. Responses were considered positive if they met the criteria described in Materials and Methods. The dotted line shows the cut off value of 0.01%.

Antigen-specific cells were identified within the CD3^+^CD4^+^ population through assessment of CD40L expression, after a short (6h) *in vitro* antigen re-stimulation. This method ensures stability of phenotypic features and allows analysis of a broader population of antigen-specific cells, compared to longer protocols based on cytokine production only [22–24]. Cells were functionally characterized by evaluating cytokine (IFN-γ, IL-2) production. An example of the gating strategy and data relative to the pre-vaccination and week 1 time points for subject 1, are shown in Fig 2A; the response kinetics up to week 8 from the same individual are shown in Fig 2B.

In the depicted subject, few TT-specific CD4^+^CD40L^+^ T cells were evident prior to vaccination, however they were clearly detected 1 week post-vaccination (0.138% after background subtraction); of these 37% produced IL-2 (0.052% of CD3^+^CD4^+^) and 42% IFN-γ (0.059% of CD3^+^CD4^+^). Similar profiles were seen for the bystander antigens PPD and *C*. *Alb*. For PPD, CD40L^+^CD4^+^ T cells comprised 0.041%, i.e ~30% of the TT response, with higher proportion of cells producing IFN-γ (75%) than IL-2 (44%). Responses to *C*. *Alb* were lower but CD40L^+^CD4^+^ IL-2-producing T cells were clearly detectable (Fig 2A). Following a contraction at week 2, TT- and PPD-specific responses remained detectable during the following weeks (Fig 2B).

Cumulative data from all five donors and complete kinetics are shown in S2 Fig. Although kinetics varied among individuals, in all subjects the increase in TT-specific responses visible one week after vaccination, was paralleled by an increase in PPD-specific responses. In subject 4, this was limited to a small increase in the number of IFN-γ-producing cells. Overall, responses to *C*. *Alb* were generally weak, with an increase in CD4^+^CD40L^+^ cells visible at week 1 in 3/5 individuals (1, 4 and 5), and an increase in IL-2^+^ cells detected only in 1/5. As in subject 1 (Fig 2A and 2B), no production of IFN-γ in response to *C*. *Alb* could be seen in the remaining subjects (S2 Fig).

In summary, vaccine-specific CD4^+^ T cell responses assessed by intracellular CD40L/cytokine staining were paralleled by bystander T cell responses, confirming the observation from IFN-γ ELISPOT. In each subject, the kinetics of the responses analyzed by the two methods were largely similar (Fig 1 and S2 Fig).

### Increase of CD4^+^ memory T cells following TT booster vaccination

**Fig 3:**
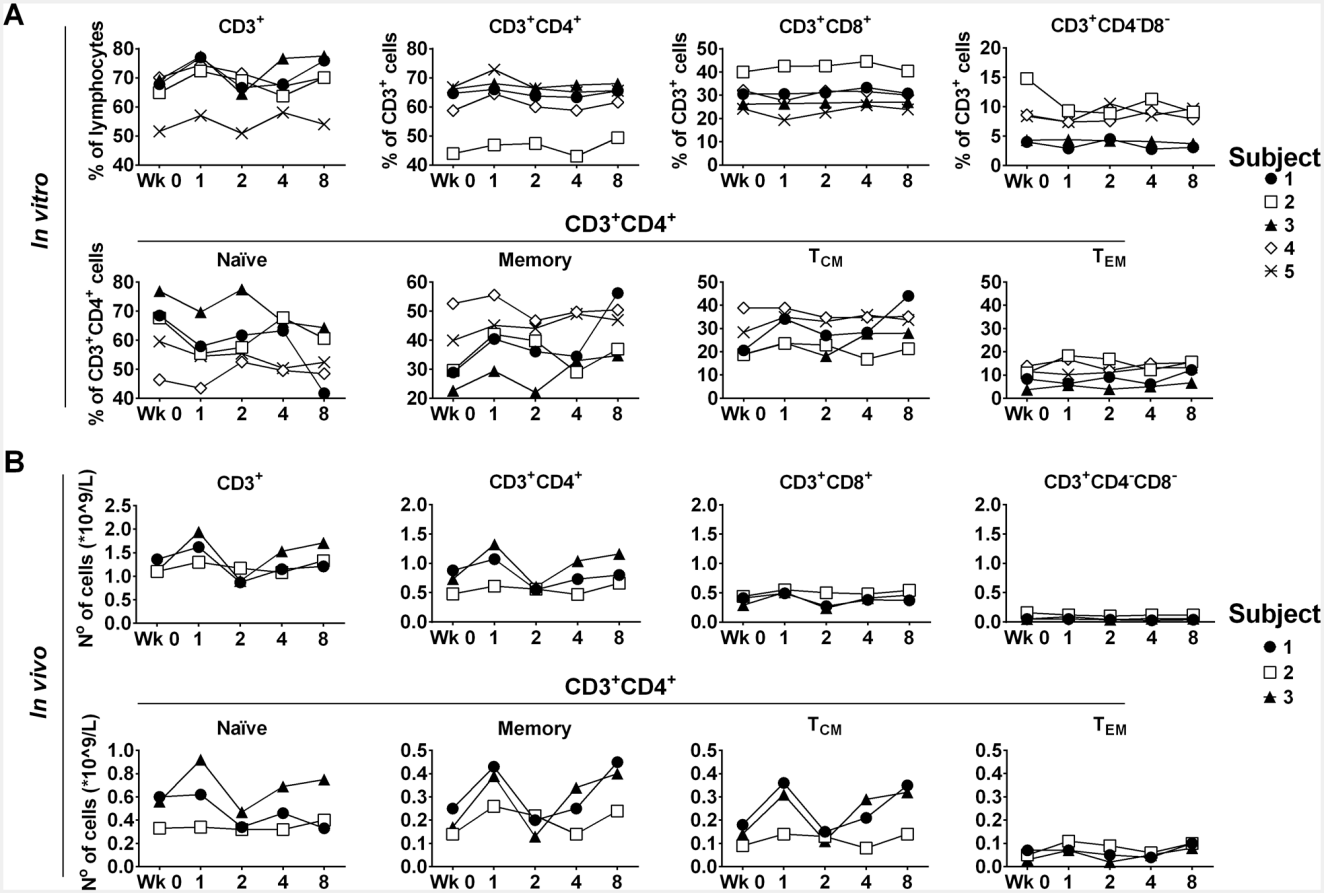
Percentages (A) and absolute numbers (B) of relevant immune cell populations before (Wk 0) and up to 8 weeks after TT booster vaccination. (A) Percentages of CD3^+^, CD3^+^CD4^+^, CD3^+^CD8^+^ and CD3^+^CD4^-^CD8^-^ cells were determined on viable PBMNC prior to cell culture by flow cytometry, gating on singlets first, and then on the lymphocyte population. A minimum of 100000 events were acquired. Combined staining for CD45RA and CCR7 allowed discrimination of Naïve (CD45RA^+^ CCR7^+^), Memory (CD45RA^-^), central memory, T_CM_ (CD45RA^-^ CCR7^+^) and effector memory, T_EM_ (CD45RA^-^ CCR7^-^) CD3^+^CD4^+^ cells. (B) The absolute number of cells was calculated from the blood lymphocyte counts available for subjects 1, 2 and 3 (Table 1), and the phenotypic data.

Since a short exposure to antigen *in vitro* was required for detection of CD4^+^ T cells, it was necessary to demonstrate the findings reflected real changes in blood precursor frequency rather than being the consequence of enhanced reactivity leading to increased cytokine production upon *in vitro* restimulation with antigen. If so, the observed changes in frequency of specific populations of CD4^+^ memory T cells should be reflected in changes in the total CD4^+^ memory T cells. Therefore, the question of whether bystander activation could lead to a rise in the total number of CD4^+^ memory T cells was addressed. Immuno-phenotyping data from cells prior to re-stimulation up to the week 8 time point are shown in Fig 3A. In all subjects, a transient increase in the proportion of CD3^+^ cells was seen at week 1 over baseline (mean increase ± SD = 6.9 ± 2%; P = 0.0015). In two cases (4 and 5), this was accompanied by an increase in the percentage of CD4^+^ T cells and relative reduction in CD8^+^ T cells. In one subject (subject 2) both CD4^+^ and CD8^+^ T cells increased with parallel reduction in CD4^-^CD8^-^ T cells. In the remaining subjects, no significant changes in the distribution of CD4^+^ and CD8^+^ T cells were observed. Among CD4^+^ T cells, a consistent increase in the proportion of memory cells (mean increase ± SD = 7.76 ± 3.65; P = 0.01) and a parallel reduction of naïve cells was evident at week 1 in all subjects (mean decrease ± SD = 7.62 ± 3.45; P = 0.01). This was accounted for mainly by a rise in central memory cells (Fig 3A). Interestingly, the smallest increase in memory cells with a relatively unchanged proportion of T_CM_ cells was seen in subject 4, who had showed the smallest increase of bystander CD4^+^ T cells as measured by intracellular CD40L/cytokine staining (S2 Fig).

White blood cell (WBC) counts were available for 3 donors (subjects 1–3) (Table 1). Total counts increased at week 1 in all subjects. In subjects 1 and 2, a rise in neutrophil counts contributed mostly to this, whilst in subject 3, neutrophils, monocytes and lymphocytes all increased. A rise in eosiniphils was also detected at week 1 in subject 1. Using the phenotypic data, absolute numbers of cells were calculated, and are reported in Fig 3B. Changes in absolute number of cells paralleled those seen in the percentage of cells, with augmented number of CD3^+^ and CD3^+^CD4^+^ visible at week 1, and among the latter, a marked increase in memory cells, particular of the central memory phenotype. Naïve cells remained stable in 2/3 individuals and increased only in subject 3, possibly as consequence of the increased number of lymphocytes (Fig 3B and Table 1).

**Table 1:** Total and differential white blood cell (WBC) counts (N x 10^9^/L) before (Week 0) and at various time points after TT booster vaccination in three healthy subjects. Full blood counts were carried out at each study visit by the routine NHS laboratory.

**Subject 1**	**Week 0**	**1**	**2**	**4**	**8**
**WBC (N x 10** ^**9**^ **/L)**	5.4	8.7	4.6	5.0	4.2
**Neutrophils**	2.5	5.4	2.5	2.6	2.2
**Eosinophils**	0.4	0.7	0.3	0.2	0.1
**Basophils**	0.1	0.0	0.0	0.0	0.0
**Monocytes**	0.4	0.5	0.5	0.4	0.3
**Lymphocytes**	2.0	2.1	1.3	1.7	1.6
**Subject 2**	**Week 0**	**1**	**2**	**4**	**8**
**WBC (N x 10** ^**9**^ **/L)**	4.4	5.0	4.7	5.2	5.0
**Neutrophils**	2.2	2.6	2.3	2.8	2.6
**Eosinophils**	0.1	0.1	0.1	0.1	0.1
**Basophils**	0.0	0.0	0.0	0.0	0.0
**Monocytes**	0.5	0.5	0.6	0.6	0.4
**Lymphocytes**	1.7	1.8	1.7	1.7	1.9
**Subject 3**	**Week 0**	**1**	**2**	**4**	**8**
**WBC (N x 10** ^**9**^ **/L)**	4.2	5.9	4.7	4.2	5.2
**Neutrophils**	2.1	2.6	2.6	1.9	2.3
**Eosinophils**	0.1	0.1	0.1	0.1	0.1
**Basophils**	0.0	0.0	0.0	0.0	0.0
**Monocytes**	0.3	0.6	0.6	0.3	0.6
**Lymphocytes**	1.6	2.5	1.4	2.0	2.2

In summary, increased numbers of white blood cells were detected one week after TT vaccination. These comprised both innate and adaptive immune cells, and within the latter, CD3^+^ CD4^+^ memory T cells appeared to be particularly increased.

### Vaccine-specific CD4^+^ T cells are mainly of effector memory type but bystander T cells are mainly central memory

**Fig 4:**
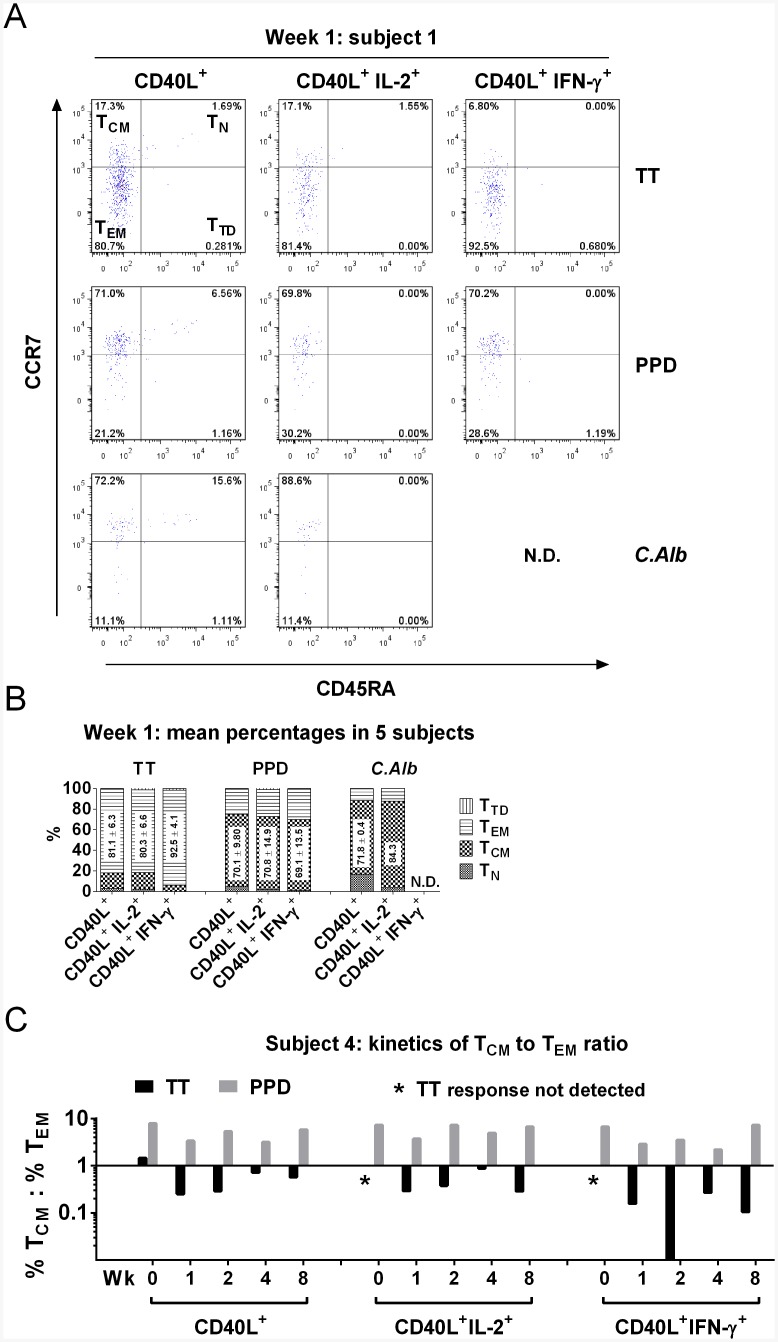
Distribution of vaccine-specific (TT) and bystander (PPD, *C*.*Alb*) CD4^+^ CD40L^+^ and cytokine positive T cells according to the expression of CD45RA and CCR7. After a short term (6h) *in vitro* culture in the absence (control) or in the presence of either TT (10μg/ml), PPD (15μg/ml) or *C*.*Alb* (10μg/ml), antigen-specific CD4^+^ T cells, identified by CD40L expression and cytokine (IL-2, IFN-γ) production (see Fig 2A), were stained with CD45RA and CCR7 and the percentages of naïve (T_N_, CD45RA^+^CCR7^+^), central memory (T_CM_, CD45RA^-^CCR7^+^), effector memory (T_EM_, CD45RA^-^CCR7^-^) and terminally differentiated (T_TD_, CD45RA^+^CCR7^-^) were calculated among the CD4^+^CD40L^+^, CD4^+^CD40L^+^IL-2^+^ and CD4^+^CD40L^+^IFN-γ^+^ cells. (A) Representative data from subject 1 showing the phenotype of vaccine-specific (TT) and bystander (PPD, *C*.*Alb*) CD4^+^ T cells one week after booster vaccination with TT. The percentages of events in each quadrant are indicated. Gates in dot plots were set using the appropriate isotype-matched controls. Responding cells are CD45RA^-^ memory type cells, but whilst the TT-specific are in their vast majority T_EM_, bystander (PPD and *C*.*Alb*-specific) cells are mainly T_CM_. N.D. Responses not detected. (B) Cumulative data (mean percentages) from five subjects, one week after TT vaccination showing the differential phenotype between vaccine-specific and bystander responses. The numbers on the bars indicate the mean percentages ± SD of T_EM_ cells among the TT-specific, and of T_CM_ cells among the PPD- and *C*.*Alb*-specific populations, respectively. *C*.*Alb*-specific CD40L^+^ cells were detected only in subjects 1, 4 and 5; *C*.*Alb*-specific CD40L^+^IL-2^+^ cells were detected only in subject 1; *C*.*Alb*-specific CD40L^+^IFN-γ^+^ cells were not detected in any subjects (N.D). (C) Kinetics of T_CM_ to T_EM_ ratio in vaccine-specific (TT) and bystander (PPD) CD4^+^ CD40L^+^ T cells in subjects 4 before TT vaccination (Wk 0) and during follow up. Black asterisks indicate time points where TT-specific responses were not detectable.

The distribution of the induced vaccine-specific and bystander CD4^+^ T cells into naïve (T_N_), T_CM_, T_EM_ and terminally differentiated (T_TD_) subsets was analyzed using CCR7 and CD45RA expression [25]. Representative dot plots at week 1 time point are shown in Fig 4A. The TT response is predominantly CD45RA negative (i.e. memory T cells) and most responding cells are CCR7 negative, indicative of effector memory type. This is consistent across the total CD4^+^CD40L^+^ population (80.7% T_EM_) as well as the IL-2^+^ and IFN-γ^+^ producing sub-populations, the latter showing an even greater proportion (92.5%) of T_EM_ cells.

The bystander response to PPD is also largely CD45RA negative; however, in contrast to the vaccine-specific, the bystander CD4^+^CD40L^+^ T cells are mainly CCR7^+^ (71%), consistent with central memory phenotype, with similar proportions of T_CM_ found across IL-2^+^ and IFN-γ^+^ sub-populations. The same distribution is also seen in cells responding to *C*. *Alb* (Fig 4A), with T_CM_ cells accounting for over 70% of the total population and almost 90% of IL-2-producing cells. *C*.*Alb*-specific IFN-γ^+^ cells were not detected. Compiled data from all subjects at week 1 are reported as mean percentages in Fig 4B and S3B Fig, as well as individually in S3A Fig. They are highly reproducible among the subjects and show the same pattern with a marked difference in the proportion of T_EM_ cells (P<0.001), which constitute the majority of the vaccine-specific population, and T_CM_ cells (P<0.001) which are preponderant among the bystander responding populations.

The kinetic of induction of T_CM_ and T_EM_ was then assessed and the ratios of T_CM_: T_EM_ cells over an 8 week period were calculated for both TT- and PPD-specific responses. Data from a representative subject and cumulative data from all the individuals are reported in Fig 4C and S4 Fig, respectively. For subjects 4 and 5, pre-vaccination T-cell responses against the two antigens were detectable, albeit at the expected low frequency, allowing comparison between pre- and post-vaccination phenotype.

Vaccination induced a shift within the TT-specific CD4^+^CD40L^+^ population, from either a T_CM_-rich (subject 4; T_CM_/T_EM_ = 1.45) or balanced (subject 5, T_CM_/T_EM_ = 0.92) distribution pre-vaccination to a post-vaccination response characterized by predominance of T_EM_ cells in both subjects (Fig 4C and S4 Fig). TT-specific IL-2 and IFN-γ-producing cells were not detectable in significant numbers in any subjects at baseline and always displayed a T_EM_ phenotype after vaccination (Fig 4C and S4 Fig). In contrast, PPD-specific responses, including cytokine-producing cells, were of T_CM_ type pre-vaccination and retained this phenotype throughout the follow up period (Fig 4C and S4 Fig).

### Vaccine-specific CD4^+^ memory T cells show reduced expression of IL-7Rα and Bcl-2, but bystander cells maintain high levels of both

**Fig 5:**
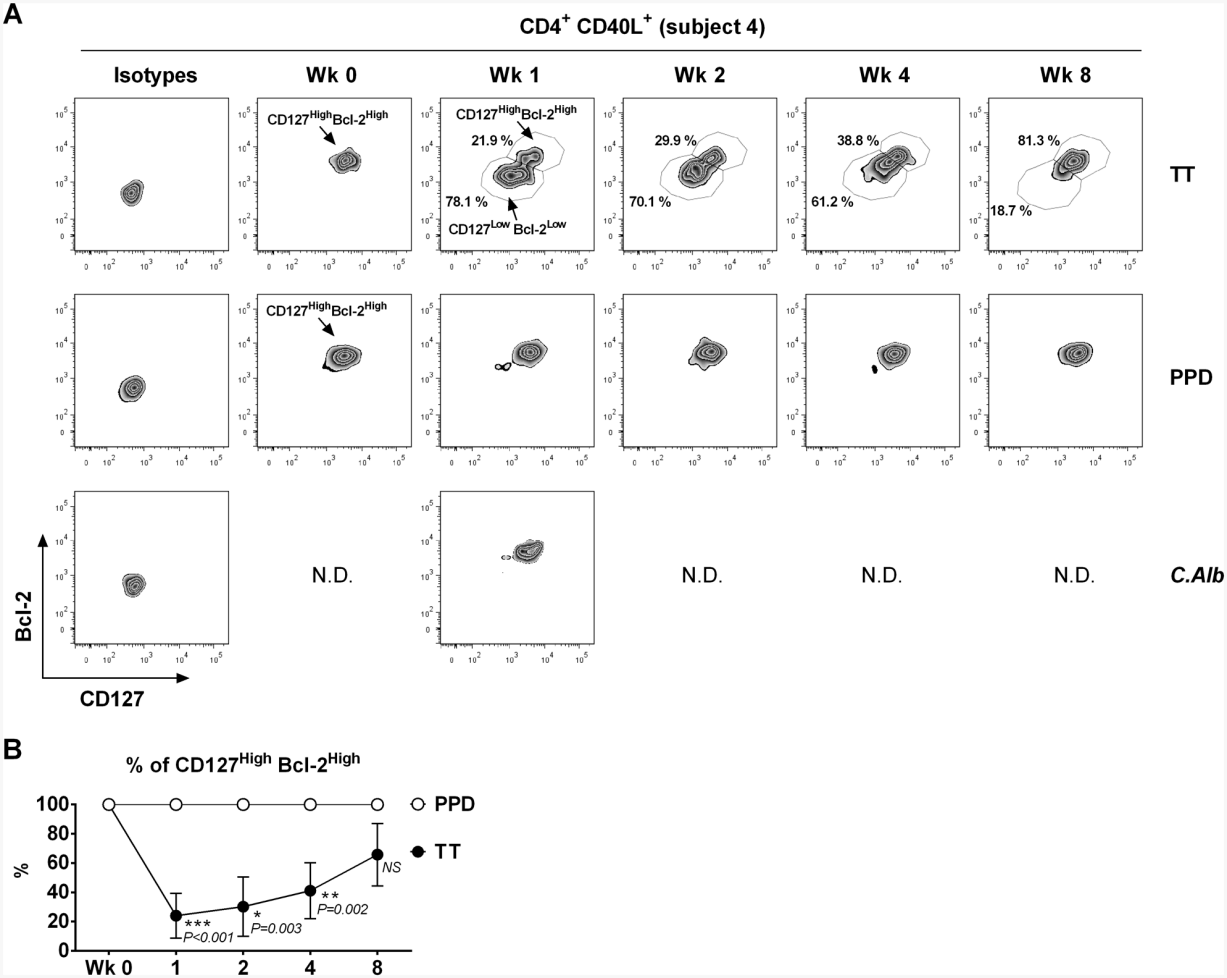
Expression of CD127 (IL-7Rα) and Bcl-2 on vaccine-specific (TT) and bystander (PPD and *C*.*Alb*) CD4^+^CD40L^+^ T cells before (Wk 0) and at various time points after TT booster vaccination. After a short term (6h) *in vitro* culture in the absence (control) or in the presence of either TT (10μg/ml), PPD (15μg/ml) or *C*.*Alb* (10μg/ml), PBMNC were first stained for surface CD3, CD4 and CD127, then permeabilized and stained intracellularly with fluorescent antibodies specific for CD40L and Bcl-2. (A) Data from a representative subject showing how expression of CD127 and Bcl-2 is reduced following TT booster vaccination on TT-specific, but not on bystander PPD-specific CD4^+^CD40L^+^ T cells. High expression of both markers is also found on *C*.*Alb*-specific cells detected at week 1. The percentage of TT-specific cells with a CD127^High^Bcl-2^High^ phenotype reaches its minimum one week after vaccination and increases again in the following weeks. (B) Cumulative data on the proportion of CD4^+^CD40L^+^ TT-specific and PPD-specific T cells with a CD127^High^Bcl-2^High^ phenotype before (Wk 0) and after TT booster vaccination. Data indicate the mean ± standard deviation calculated from the individuals showing detectable responses. Friedman paired analysis confirmed significant variance across time course for TT-specific population (P = 0.0031); individual P-values given at each time point generated using paired T-test comparing to baseline. At week 0, responses to TT and PPD were detected in subjects 4 and 5; at week 2, responses to PPD were detected in subjects 2 and 4; for all the remaining time points, responses to TT and PPD were detected in all subjects.

Expression of IL-7Rα (CD127) and the anti-apoptotic Bcl-2 tend to decrease in recently-generated effector CD4^+^ [26] and CD8^+^ [21] T cells. We therefore measured expression of CD127 and Bcl-2 on vaccine-specific and bystander CD4^+^CD40L^+^ T cells. Data over the 8 week follow up period are shown for a representative subject in Fig 5A, and cumulative data for the remaining subjects at week 1 are depicted in S5 Fig. Pre-vaccination TT-specific cells were CD127^High^Bcl-2^High^. One week after vaccination, a significant proportion (median 78.1%, range 50.0–90.1%; P < 0.001) showed reduced expression of both markers. The percentage of CD127^High^Bcl-2^High^ cells within the TT-specific CD4^+^CD40L^+^ population slowly increased in the following weeks (Fig 5A), but remained significantly reduced relative to baseline until week 8 (Fig 5B).

In contrast, PPD-specific CD4^+^ CD40L^+^ T cells were also CD127^High^Bcl-2^High^ at baseline, but they maintained this phenotype one week after vaccination and at later time points (Fig 5A and S5 Fig). The *C*.*Alb* specific response detected at week 1 was also characterized by cells uniformly high in CD127 and Bcl-2 (Fig 5A and S5 Fig).

Kinetic analysis (mean percentages ± SDEV) of CD127^High^Bcl-2^High^ cells in all subjects is displayed in Fig 5B and it shows the significant changes from baseline in the TT-specific CD4^+^CD40L^+^ population and the remarkable stability of PPD-specific cell phenotype.

### Vaccine-specific but not bystander CD4^+^ memory T cells display high levels of activation and proliferative activity

**Fig 6:**
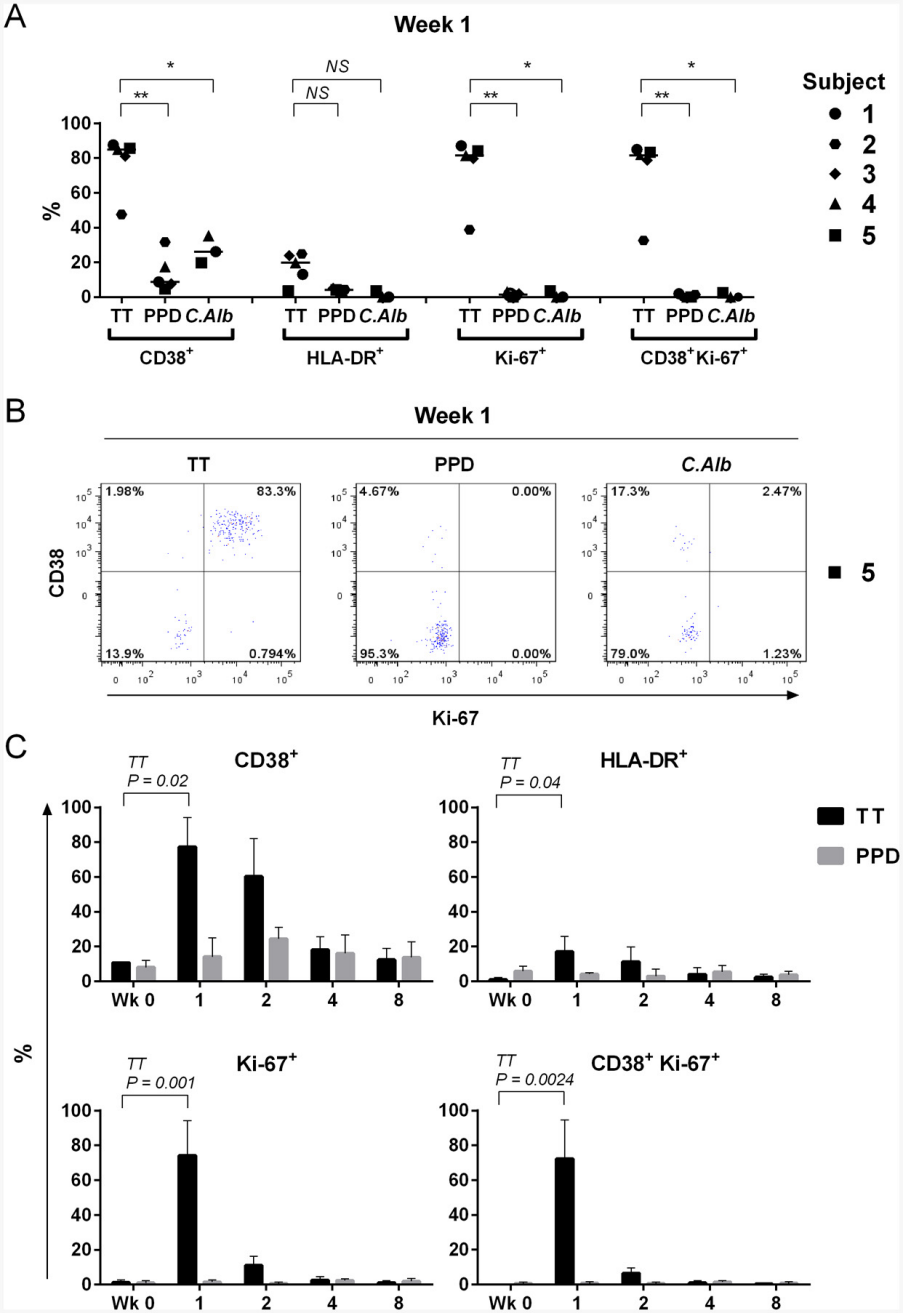
Analyis of activation (CD38, HLA-DR) and proliferation (Ki-67) markers on vaccine-specific (TT) and bystander (PPD and *C*.*Alb*) CD4^+^CD40L^+^ T cells. After a short term (6h) *in vitro* culture in the absence (control) or in the presence of either TT (10μg/ml), PPD (15μg/ml) or *C*.*Alb* (10μg/ml), PBMNC were first stained for surface CD3, CD4, CD38 and HLA-DR, then permeabilized and stained intracellularly for CD40L and Ki-67. (A) Data indicate the percentage of CD38, HLA-DR, Ki-67 single positive and CD38^+^Ki-67^+^ double positive cells within the CD3^+^CD4^+^CD40L^+^ population in five healthy subjects one week after receiving a booster vaccination with TT. Median values are indicated by a bar. Significance of the difference between vaccine specific and bystander populations are indicated (NS—non-significant, * = P<0.01, ** = P<0.001), as analysed using non-parametric Mann-Whitney U-test. (B) Representative dot plots from subject 5, showing how at week 1 activated and proliferating (CD38^+^Ki-67^+^) cells are only found among the TT-specific cells, but not among the bystander PPD- and *C*.*Alb*-specific cells. Percentages of positive cells in the CD3^+^CD4^+^CD40L^+^ gated population are indicated in each quadrant. Gates in dot plots were set using the appropriate isotype-matched controls. (C) Analysis of expression (%) of CD38, HLA-DR, Ki-67 single positive and CD38^+^Ki-67^+^ double positive cells within the TT- and PPD-specific CD4^+^CD40L^+^ T cell populations before (Wk 0) and at various time points after TT booster vaccination. Data indicate the mean ± standard deviation calculated from the individuals showing detectable responses. P-values indicate the significant changes in expression over baseline for all time points, calculated using paired T-test and confirmed using Friedman’s test; non-significant changes are unlabeled. At week 0, responses to TT and PPD were detected in subjects 4 and 5; at week 2, responses to PPD were detected in subjects 2 and 4; for all the remaining time points, responses to TT and PPD were detected in all subjects.

Activation status and *in vivo* proliferative activity of CD4^+^CD40L^+^ antigen-specific T cells were investigated by analyzing expression of surface markers CD38 and HLA-DR, and intracellular Ki-67, respectively (Fig 6). At week 1, a high percentage of vaccine-specific (TT) cells expressed CD38 (median 84.9%, range 47.5–87.5%), and a proportion of these were HLA-DR^+^ (median 19.8%, range 3.5–24.8%). Importantly, the majority were also Ki-67^+^ (median 81.5%, range 38.7–87.0%) (Fig 6A). This indicates recent or on-going activation and proliferative activity. Conversely, PPD- and *C*.*Alb*-specific CD4^+^CD40L^+^ T cells, despite showing some degree of CD38 expression (medians 8.7 and 26.1%, ranges 4.6–31.6 and 19.7–35.3%, respectively), remained HLA-DR negative and Ki-67 negative. Hence, whilst double positive (CD38^+^Ki-67^+^) cells constituted the majority of TT-specific cells, they were virtually undetectable among the bystander populations (Fig 6A and 6B).

Analysis of expression (mean % ± SDEV) of these markers in TT- and PPD-specific CD4^+^CD40L^+^ T cells pre- and post-vaccination is shown in Fig 6C. Clearly, the presence of activated and proliferating cells (CD38^+^Ki-67^+^) among the vaccine-specific cells was transient. They were absent at baseline, they increased significantly one week after vaccination (mean% TT reactive cells expressing markers ± SD = 72.2 ± 22.3; P = 0.0024), and then rapidly declined, with numbers at week 2 not significantly different to those at baseline (5.1 ± 4.0). This was attributable to the rapid loss of Ki-67^+^ expression on cells (at week 2 only (8.8 ± 6.7) of TT-specific cells were still positive), whereas persistence of CD38+ expression on TT-specific cells at week 2 was more varied across the cohort (60.3 ± 21.7. Range 31.0–79.0).

PPD-specific CD4^+^CD40L^+^ T cells, despite showing a small, non-significant increase in CD38 expression following vaccination, always remained Ki-67 negative throughout the follow up period.

## Discussion

Dissecting the mechanisms responsible for the maintenance of T cell memory has major repercussions on vaccine development. We have previously reported in a larger study [12], and confirmed here, that following TT recall vaccination, the Th_mem_ immune response to TT, is accompanied by an increase of vaccine unrelated Th_mem_ cells. This phenomenon fits with the view that there might be a “tick-over” mechanism acting globally on memory T cells so that each time there is an infection, memory gets a stimulus, and this may contribute to memory maintenance. Other circumstantial observations of parallel increase of heterologous immunity following TT vaccination [27,28], and vice-versa, expansion of TT-specific CD4^+^ T cells during an episode of common influenza virus infection [29], support this view.

To clarify the nature of the bystander cells and the mechanisms driving their increase in the peripheral blood, we first addressed their phenotypic and functional features and we compared them to those of vaccine-specific CD4^+^ memory T cells.

Analysis of the responses at the pre-vaccination baseline, revealed similarities between the TT-specific and bystander (PPD-specific) CD4^+^ CD40L^+^ T cells: both were CD45RA^-^CCR7^+^ (T_CM_), expressed high levels of CD127 and Bcl-2 and were not activated and non-proliferating. At the peak of the response to vaccination (week 1), key differences emerged. The TT-specific CD4^+^ T cells displayed the typical features of recently generated effector T cells. They were CCR7^-^ (T_EM_), mainly CD127^Low^Bcl-2^Low^, and showed clear signs of activation (CD38^+^HLA-DR^+^) and proliferative activity (Ki-67^+^). This allows delineation of the response to vaccination within a naturally fluctuating immune activity in human subjects. In contrast, both PPD- and *C*.*Alb-*specific CD4^+^ T cells maintained expression of CCR7 (T_CM_), CD127 and Bcl-2, and crucially, showed no evidence of activation (HLA-DR^-^ CD38^Low^) or proliferation (Ki-67^-^). These differences were consistent in all the subjects.

To our knowledge, this is the first study to provide a detailed characterization of vaccine-specific and bystander CD4^+^ memory T cells in response to protein vaccination and to identify their distinct functional and phenotypic features. Cellerai et al. [26] studied phenotype and function of vaccine-specific, but not bystander, CD4^+^ T cells before and after TT recall vaccination; they focused the analysis on cytokine-producing cells following 16h *in vitro* re-stimulation with high antigen dose (100μg/ml TT). Here, conditions of minimal stimulation *in vitro* (6h, 10μg/ml TT) were designed to assess cell status *in vivo* by avoiding perturbation of T cell phenotype and function; furthermore, CD40L expression analysis, allowed access to a wider population of antigen-specific cells, including antigen-specific cells with non-immediate effector functions or with the potential to produce cytokines not commonly evaluated. Despite these methodological differences, in both studies, the phenotypic characterization of TT-specific responses produced similar results.

With regard to bystander CD4^+^ memory T cells, the increased number detected one week after vaccination, seems to reflect a real increase in precursor frequency, as evidenced by the rise in the absolute number of circulating total CD4^+^ memory T cells (Fig 3), and of T_CM_ cells in particular. A similar apparent increase of non-proliferating CD4^+^ memory T cells specific for TT was observed in healthy subjects undergoing primary vaccination with the yellow fever vaccine [30], but no further phenotypic characterization of those cells was performed.

Expression of CCR7 on bystander cells is expected to confer the ability to re-circulate through secondary lymphoid organs. Hence, these expanded cells may have originated from cells exposed to the cytokine-rich microenvironment created in vaccine-draining lymph nodes by the secondary immune response to TT vaccination. Nonetheless, the lack of expression of activation and proliferation markers observed at the peak of the vaccine-specific response (week 1) and afterwards suggests that this is not the case. It appears more likely that the increase in circulating bystander cells is the result of mobilization and migration into the blood stream of memory cells resident in other compartments. Human CD4^+^ memory T cells residing in both lymphoid and non-lymphoid (mucosal) tissues have been recently characterized [31]. They constitutively express the T cell activation marker CD69, which is not expressed on their circulating counterpart, and are CD127 (IL-7Rα) ^+^. In the mouse, CD69 is required for the relocation of CD4^+^ T cells from the blood to the bone marrow and their persistence there [32]. Human bone marrow is enriched in polyfunctional CD4^+^ memory T cells that despite being CD69^+^ are in a resting state, as indicated by lack of expression of Ki-67, and by cell cycle and transcriptome analysis [33]. Furthermore, they express CD127 and appear protected from apoptosis [33]. We did not investigate CD69 expression, as this marker would be promptly up regulated following the *in vitro* re-stimulation with antigen required to identify antigen-specific CD4^+^ T cells in our system. However, all the phenotypic feature of vaccine-stimulated bystander CD4^+^ memory T cells appear to match those of the cells described above. In our study, the bystander Th_mem_ cells appeared non-activated and non-proliferating, but retained high expression of Bcl-2, indicative of preserved survival potential. Furthermore, they displayed potential effector functions, as demonstrated by the prompt production of cytokines after brief *in vitro* re-stimulation with cognate antigen. Considering these similarities, it is therefore conceivable that the bystander cells we detected after TT vaccination may derive from CD4^+^ memory T cells resident in tissues, possibly the bone marrow, and mobilized following the recall vaccination. Interestingly, the increase of non-vaccine specific Th_mem_ cells we describe here, resembles that seen in the same vaccination setting, of non-TT specific plasma cells. These appeared to be mobilized from survival niches in the bone marrow by either competing newly formed TT-specific plasma blasts [34] or possibly by the disruptive action of adjuvant (alum) stimulation [35]. To understand the role of alum in relation to the increase of bystander CD4^+^ memory T cells, administration of alum as single agent would be required, but it is unlikely to be ethically justifiable in humans.

In summary, it appears that following vaccination or infection, a pool of CD4^+^ memory T cells are mobilized from depot tissues (e.g. the bone marrow) and enter the blood stream. This perhaps is part of a response aimed at speeding up and raising defence against other opportunistic pathogens. As the mobilized cells express CCR7, they could reach secondary lymphoid organs, where the putative pathogen-derived antigens would in the meantime been transferred and presented to them.

The clear phenotypic distinction between vaccine-specific and vaccine-stimulated bystander CD4^+^ memory T cells we provide here has not only biological but also important practical implications for vaccinology. The increase of bystander CD4^+^ memory T cells following vaccination poses problems to a correct evaluation of vaccine specific responses, because an increase in vaccine-specific CD4^+^ memory T cells may result from their mobilization as a bystander response to a different immunogen, rather than from specific proliferation to the vaccine. We provide here the tools to integrate and validate results obtained by the quantitative analysis of blood precursor frequency, to contribute to more accurate evaluation of the CD4^+^ T cell response to vaccination in humans.

These findings reveal the dynamic nature of the T-cell arm of the human immune response which underlies responses to environmental perturbations, measurement of immune responses and disturbances in hematologic diseases.

## Supporting Information

S1 Fig Kinetics of vaccine-specific and bystander T cell responses to TT recall vaccination measured by a 40h IFN-γ ELISPOT assay, using freshly isolated PBMNC. The assay was set up within two hours of blood collection. Responses are reported as the mean number of spots ± SD of triplicate antigen-stimulated cultures, after subtracting the correspondent negative (no antigen) control.
(TIF)

S2 Fig Kinetics of vaccine-specific and bystander CD4^+^ T cell responses detected by intracellular CD40L and cytokine staining in five healthy subjects, who received a booster vaccination (Wk 0) with TT. After a short term (6h) *in vitro* culture in the absence (control) or in the presence of either TT (10μg/ml), PPD (15μg/ml) or *C*.*Alb* (10μg/ml), PBMNC were first stained for surface CD3 and CD4, then permeabilized and stained intracellularly with fluorescent antibodies specific for CD40L and the cytokines IL-2 and IFN-γ. Data indicate the frequency of positive cells within the CD3^+^CD4^+^ population, obtained from the antigen-stimulated samples after subtracting the frequency of events in the control (no antigen) cultures. Responses were considered positive if they met the criteria described in Materials and Methods. The dotted line in each graph shows the cut off value of 0.01%.
(TIF)

S3 Fig Distribution of vaccine-specific (TT) and bystander (PPD, *C*.*Alb*) CD4^+^ CD40L^+^ and cytokine positive T cells according to the expression of CD45RA and CCR7. Five healthy subjects received a booster vaccination of TT. PBMNC were cultured *in vitro* for 6h in the absence (control) or in the presence of either TT (10μg/ml), PPD (15μg/ml) or *C*.*Alb* (10μg/ml) and the distribution of naïve (T_N_, CD45RA^+^CCR7^+^), central memory (T_CM_, CD45RA^-^CCR7^+^), effector memory (T_EM_, CD45RA^-^CCR7^-^) and terminally differentiated (T_TD_, CD45RA^+^CCR7^-^) was studied among the vaccine-specific and bystander CD4^+^CD40L^+^, CD4^+^CD40L^+^IL-2^+^ and CD4^+^CD40L^+^IFN-γ^+^ cells, one week after vaccination. Responding cells are CD45RA^-^ memory type cells, but whilst the TT-specific are in their vast majority T_EM_, bystander (PPD and *C*.*Alb*-specific) cells are mainly T_CM_. (A) Individual data. N.D. Responses not detected. (B) Summary data for proportional representation of memory subtypes within the antigen specific CD4^+^ T cell populations identified by either CD40L^+^, CD40L^+^IFNγ^+^ or CD40L^+^IL-2^+^; proportional representation within vaccine-specific and bystander populations compared using T-test with Holm-Sidak adjustment of P value to take account of multiple testing. Significant differences highlighted (**) alongside given P-value.
(TIF)

S4 Fig Kinetics of T_CM_ to T_EM_ ratio in vaccine-specific (TT) and bystander (PPD) CD4^+^ T cells in 5 healthy subjects before TT vaccination (Wk 0) and during follow up. PBMNC were cultured for 6h *in vitro* in the absence (control) or in the presence of either TT (10μg/ml) or PPD (15μg/ml) and the ratio between percentages of central memory (T_CM_, CD45RA^-^CCR7^+^) and effector memory (T_EM_, CD45RA^-^CCR7^-^) was calculated among the total (CD40L^+^) and the cytokine (IL-2 and IFN-γ)-producing CD3^+^CD4^+^ T cells. Black asterisks and gray stars indicate time points where TT-specific and/or PPD-specific responses respectively were not detectable.
(TIF)

S5 Fig Analysis of CD127 and Bcl-2 expression on vaccine-specific (TT) and bystander (PPD and *C*.*Alb*) CD4^+^CD40L^+^ T cells, one week after booster vaccination with TT. After a short term (6h) *in vitro* culture in the absence (control) or in the presence of either TT (10μg/ml), PPD (15μg/ml) or *C*.*Alb* (10μg/ml), PBMNC were first stained for surface CD3, CD4 and CD127, then permeabilized and stained intracellularly with fluorescent antibodies specific for CD40L and Bcl-2. Cumulative data showing the appearance of a population among the TT-specific but not among PPD- and *C*.*Alb*-specific CD4^+^CD40L^+^ T cells, characterized by low expression of CD127 and Bcl-2. N.D. Responses not detected.
(TIF)
